# A Low-Cost Microfluidic Method for Microplastics Identification: Towards Continuous Recognition

**DOI:** 10.3390/mi13040499

**Published:** 2022-03-23

**Authors:** Pedro Mesquita, Liyuan Gong, Yang Lin

**Affiliations:** Department of Mechanical, Industrial and Systems Engineering, University of Rhode Island, Kingston, RI 02881, USA; pedro_mesquita@uri.edu (P.M.); liyuan_gong@uri.edu (L.G.)

**Keywords:** microfluidics, microplastics, continuous identification, low-cost, 3D printing

## Abstract

Plastic pollution has emerged as a growing concern worldwide. In particular, the most abundant plastic debris, microplastics, has necessitated the development of rapid and effective identification methods to track down the stages and evidence of the pollution. In this paper, we combine low-cost plastic staining technologies using Nile Red with the continuous feature offered by microfluidics to propose a low-cost 3D printed device for the identification of microplastics. It is observed that the microfluidic devices indicate comparable staining and identification performance compared to conventional Nile Red staining processes while offering the advantages of continuous recognition for long-term environmental monitoring. The results also show that concentration, temperature, and residency time possess strong effects on the identification performance. Finally, various microplastics have been applied to further demonstrate the effectiveness of the proposed devices. It is found that, among different types of microplastics, non-spherical microplastics show the maximal fluorescence level. Meanwhile, natural fibers indicate better staining quality when compared to synthetic ones.

## 1. Introduction

It has been estimated that the ongoing COVID-19 pandemic has exceedingly increased the demand for the use of plastics [1]. As of 23 August 2021, more than 8.4 million tons of pandemic-associated plastic debris were released into the oceans [2]. Among them, most of the plastic debris was microplastics with a size smaller than 5 mm [3,4], and this can be classified into primary and secondary microplastics. Generally, primary microplastics are the microscopic plastics that were intentionally made small (e.g., microbeads used in cosmetics) [5,6,7], while secondary microplastics are particles resulting from the breakdown of macroscopic pieces due to the conjoint environmental effects (i.e., photo-oxidation, hydrolysis, microorganism degradation, mechanical shear, etc.) [8,9]. Despite the fact that a variety of plastic types have been identified in microplastics, most of the microplastics in seawater originate from packaging materials (e.g., polyethylene, and polypropylene) [10]. Owing to their small density, these microplastics tend to float on the surface of the water, thus they can spread worldwide (in opposition to denser plastics that tend to settle down) and are more difficult to remove [11,12].

Nevertheless, the current understanding of plastic pollution in terms of the quantity, type, lifetime, and associated health effects largely remains unknown. As a result, microplastic separation and identification serve as an important approach to providing evidence and metrics of the pressing environmental issues caused by plastic pollution [13]. For example, worldwide microplastic assessment is possible to identify hot pollution spots and determine the historical trends which may lead to novel strategies for fighting debris spread [14,15,16]. At present, quite a few identification techniques have been explored for microplastic identification. Among them, common methods include visual inspection, Fourier-transform infrared spectroscopy (FTIR), Raman spectroscopy and scanning electron microscopy (SEM) [17,18]. Despite their effectiveness, these techniques, except visual inspection, rely on expensive apparatus and time-consuming detection methods that are limited to trained personnel, thus hindering the expansion of these methods for high-throughput detection [19,20,21].

As a result, visual inspection, though not as effective as other sophisticated counterparts, is still widely applied for faster recognition [22,23]. Currently, a variety of sampling and identification technologies have been used to improve the performance of visual inspection, of which a commonly used one is the combination of filtration and staining [24,25]. However, filtration often leads to false positives due to potential interference from organic matters in the samples [26,27]. More importantly, its performance is highly reliant on the size of the filters, thus limiting its capabilities in sampling small microplastics. In addition, particles are also prone to adhere to the filters, resulting in ineffective separation of the microplastics for identification [28,29]. In addition, staining of the microplastics relies on staining agents that turn microplastics into prominently visible particles [30,31]. Currently, it is unsurprising that quite a few staining agents (e.g., Rhodamine B, Rose Bengal, Trypan Blue, etc.) have been explored for this purpose. Among them, Nile Red is a hydrophobic fluorophore and was reported to be one of the most effective agents due to its favorable binding performance with lipophilic substances [30,32,33].

Nevertheless, the focus of current studies on microplastic staining and identification has been largely given to batch-by-batch or case-by-case analysis [25,34,35,36]. Therefore, sample collection is still inevitable and remains a time-consuming step in the whole sampling process. Moreover, temporal information is hardly achievable. Given the need of acquiring in-depth studies of microplastic pollution in oceans and other water bodies, long-term monitoring or continuous monitoring is essential and a low-cost, simple and effective method should be developed. Thanks to the burgeoning developments of microfluidic technologies over the past decade, microfluidic devices can be a promising solution to address this need due to their powerful particle control capabilities and the ease of integration in modern electronic systems [37,38]. For example, microfluidics has been used for long-term monitoring of algae in the past [39]. Tumor responses to hypoxia conditions were also analyzed continuously in a microfluidic platform [40]. Indeed, microplastic identification is also not a new research area for microfluidics [41,42], yet to the best knowledge of the authors, microfluidics has not been applied for long-term microplastics assessment and we believe the combination of low-cost Nile Red staining and microfluidic fluid control would provide a novel venue to confront the ever-deteriorating plastic issues without complicated analysis and costly instrumentations. Low-cost fabrication methods such as 3D printing and molding can be applied to further minimize the cost associated with this method, and the miniaturized devices may also be integrated into the monitoring stations near seashores, along with data collection of other water quality metrics on a continuous basis.

**Figure 1:**
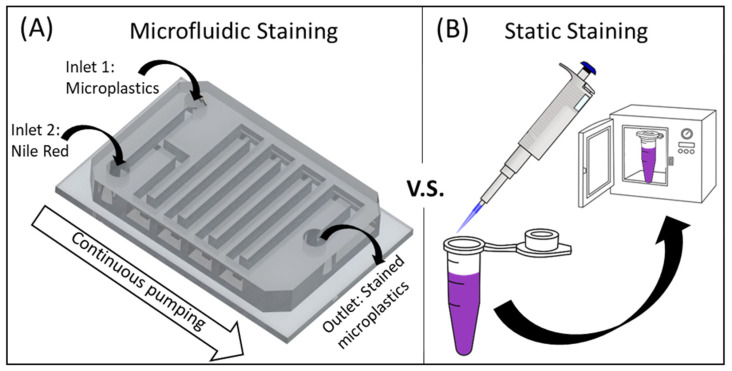
Schematic illustration of the staining processes studied in this paper. (**A**) Process of microfluidics-based continuous staining of microplastics using Nile Red. (**B**) Process of static staining of microplastics. Compared to microfluidic staining, the static process is laborious as it requires multiple batches and manual operation.

Herein, we further explored the staining capabilities of Nile Red through a microfluidic device capable of continuously staining microplastics for rapid identification. The proposed device has two inlets for respective Nile Red and sample injection (Figure 1A), and a serpentine channel that allows for sufficient mixing of the staining agent and the sample. In this paper, we studied the effects of dominating parameters on the identification performance, including Nile Red concentration, temperature, and residency time. Note that prior to performing microfluidic studies, static studies that resemble traditional Nile Red staining processes were adopted and served as a baseline for comparison.

## 2. Materials and Methods

In this paper, the process of static microplastic identification using Nile Red was carried out without a filter. Specifically, the staining Nile Red solution was added directly into an Eppendorf tube containing the microplastics sample and placed inside an oven (Figure 1B). On the other hand, microfluidic experiments followed a similar procedure: mixing Nile Red and microplastics in the device (which was placed inside an oven). Since the mixing process is passively induced without human operation, this process holds promise for continuous staining.

### 2.1. Nile Red Preparation

The staining solution was prepared by dissolving Nile Red (technical grade, N3013, Sigma-Aldrich, St. Louis, MO, USA) in methanol to different concentrations. We have considered the limit of solubility of Nile Red in methanol (1 mg/mL) to be the stock solution for further dilution, from which the solution was diluted into 50X, 100X, 250X, 500X, and 1000X samples.

### 2.2. Microplastics Sample Preparation

In this paper, lab-prepared and commercially available microplastics were adopted in lieu of naturally formed microplastics. More specifically, microspheres made of polyethylene (PE), ranging from 10–45 µm (Cospheric, Inc., Santa Barbara, CA, USA) were applied to determine the optimal parameters for staining. Other plastics including the microspheres made of polystyrene (PS) with sizes from 9.5–11.5 µm (Cospheric, Inc., Santa Barbara, CA, USA), cotton and acrylic fabric acquired from clothing, polypropylene (PP) and non-spherical PE prepared from plastic storage containers were also applied to test the versability of the proposed method. All the samples were mixed with deionized (DI) water prior to staining. Note that commercial microspheres were diluted in a concentration of 10 mg/mL, while the other samples were diluted to 1 mg/mL, which is because the commercial particles were more available than the ones obtained from other sources.

### 2.3. Static Experiments

To prepare the samples for static experiments, 100 µL of Nile Red solution was thoroughly mixed with 100 µL of PE microplastic solution inside an Eppendorf tube, followed by baking inside an oven (Quincy Lab, model 10, Burr Ridge, IL, USA). On the other hand, all static experiments were performed using PE microspheres. To investigate the effect of Nile Red concentration on staining performance, different concentrations were tested: 100X, 250X, 500X, and 1000X; in addition, different temperatures (i.e., 25, 40, 50, 60, 70, and 80 °C) were applied to study the effects of temperature. All the samples were placed inside the oven for 10 min, and analysis was conducted immediately after baking.

### 2.4. Microfluidic Experiments

To create the microfluidic devices, soft lithography, a commonly used method in microfluidics, was applied. Specifically, a 3D printer (CADWorks 3D, µMicrofluidics edition, Toronto, ON, Canada) was used to create the molds for casting polydimethylsiloxane (PDMS) to obtain the final devices. After curing the PDMS mixture in an oven overnight at 65 °C, a corona treater (BD-20AC Laboratory Corona Treater, Electro-Technic Products, Chicago, IL, USA) was used to permanently bond the device onto a glass slide. Finally, the device was placed inside the oven and a syringe pump (Fusion 200, Chemyx Inc., Stafford, TX, USA) was used to run the samples as well as the staining agents inside the device.

### 2.5. Sample Observation

For both static and microfluidic staining, an inverted microscope (Zeiss Axio Vert.A1) was used. To visualize the fluorescent signal from the samples, an illumination system (X-Cite mini+, Excelitas, Waltham, MA) with a wavelength of 365 nm was used. All the images were recorded using a camera attached to the microscope (VEO E310L, Phantom, Wayne, NJ, USA). ImageJ (https://imagej.nih.gov/ij/, accessed on 10 November 2021) was used to analyze and quantify the results. Each experiment was performed four times for statistical analysis. We have not filtered the particles prior to observation, instead, we have directly placed a droplet of the diluted sample on top of a glass slide.

## 3. Results

### 3.1. Static Results

**Figure 2:**
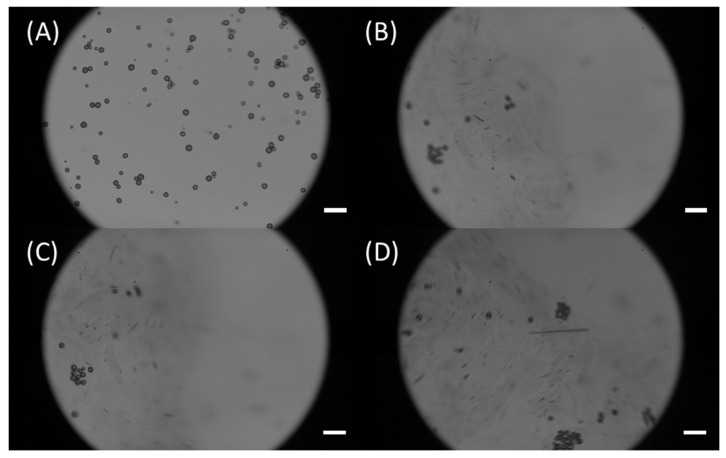
Aggregation induced due to high Nile Red concentrations. (**A**) First frame (0.0003 s)—Moment in which the Nile Red solution is placed on the glass slide right after the preparation; (**B**) Second frame (0.0006 s)—Beginning of the aggregation; (**C**) Third frame (0.0010 s)—Initial particle clusters can be observed; (**D**) Fourth frame (0.0013 s)—Higher levels of aggregation are observed; Scale bars are 100 µm.

It is worth mentioning that high concentration Nile Red can lead to undesired aggregation [43,44], which may clog microfluidic channels and mask signals from stained microplastics. Moreover, the aggregation may destroy the samples into unrealistic microplastics (once aggregated the original size and shape are lost) and induce misleading conclusions [45,46]. We have observed that aggregations occurred for Nile Red solutions diluted up to 50X. Therefore, Nile Red solutions diluted to a minimum of 100X were used in our experiments to guarantee that no induced aggregations would happen. Figure 2 illustrates how aggregation occurs over time. Specifically, 50X Nile Red solution was placed onto a glass slide containing PE microspheres, and the aggregation process was recorded at 3000 fps. Figure 2A shows the initial frame (0.0003 s), it is possible to observe that particles are separated. The other images show the subsequent frames (from 0.0006 s to 0.0013 s), where the aggregation is shown. In this image, it is possible to see how fast aggregations are induced in microplastics due to the excess of Nile Red. Furthermore, the original features of the particles are lost, if someone were to study the size distribution or the shape of this sample, the outcome would certainly not be accurate due to the aggregation.

**Figure 3:**
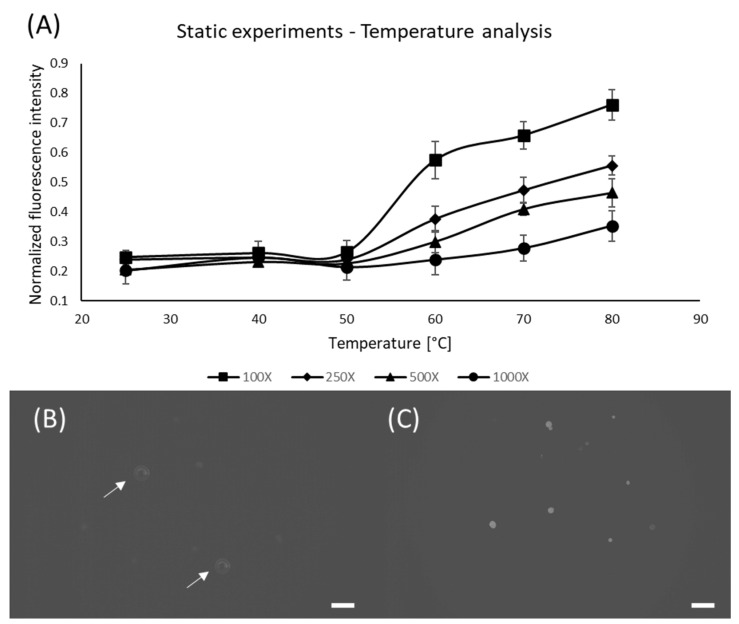
Effect of Nile Red concentration and temperature for static samples placed inside the oven for 10 min. (**A**) Graph showing the influence of different Nile Red concentrations and oven temperatures on staining performance; (**B**) PE microspheres stained using 100X Nile Red at 25 °C; (**C**) PE microspheres stained using 100X Nile Red at 80 °C; Arrows were added to indicate the particles of interest. Scale bars are 100 µm.

Once the threshold for the Nile Red concentration was defined, static experiments were conducted to determine the effects of Nile Red concentration, and temperature on staining efficiency. It was already known that temperature, residency time and ambient lights were important for the staining quality, however, no systematic study was available [30,47]. We have observed that for an infinitely long time (72 h) the highest pixel intensity of a sample containing 100X Nile Red at 25 °C is 150, thus we defined this intensity to be the reference for results normalization (all results shown in this paper are normalized with respect to this result). Figure 3 shows the results for the concentration and temperature static analysis, indicating that higher concentrations associated with higher temperatures provide better staining results, which is in accordance with the results from other groups [47,48]. However, it is difficult to identify relevant fluorescent signals at 25 °C, thus we have added arrows to indicate the particle positions.

**Figure 4:**
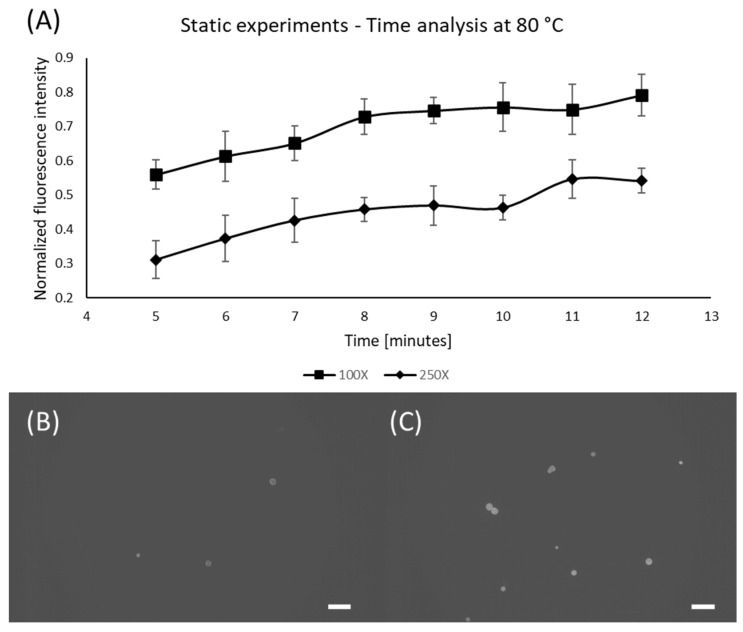
Effect of time for 100X and 250X Nile Red solutions at 80 °C. (**A**) Graph showing the influence of time; (**B**) PE microspheres stained using 100X Nile Red for 5 min; (**C**) PE microspheres stained using 100X Nile Red for 12 min; Scale bars are 100 µm.

Following the concentration and temperature experiments, we determined the effect of time on the staining quality (Figure 4). To do so, samples were kept inside the oven at a fixed temperature and Nile Red concentration, varying only the time. Since the previous results indicate that 100X and 250X Nile Red solutions at 80 °C are the most prominent combinations, thus these parameters were chosen along with variation in time: 5, 6, 7, 8, 9, 10, 11, and 12 min. As shown in Figure 4A, after 10 min, no significant changes in fluorescence level were observed, which means that this is enough time to extract the maximum performance from the staining agent. Figure 4B,C show the differences between the minimum and maximum staining time, where it is possible to observe that more time produces a stronger fluorescence signal in the particles.

### 3.2. Microfluidic Results

As aforementioned, microfluidics hold great potential in providing continuous monitoring of microplastics in various water bodies. In this section, we applied the parameters under optimal conditions obtained from static experiments to explore the possibilities of using microfluidics for continuous microplastic identification.

**Figure 5:**
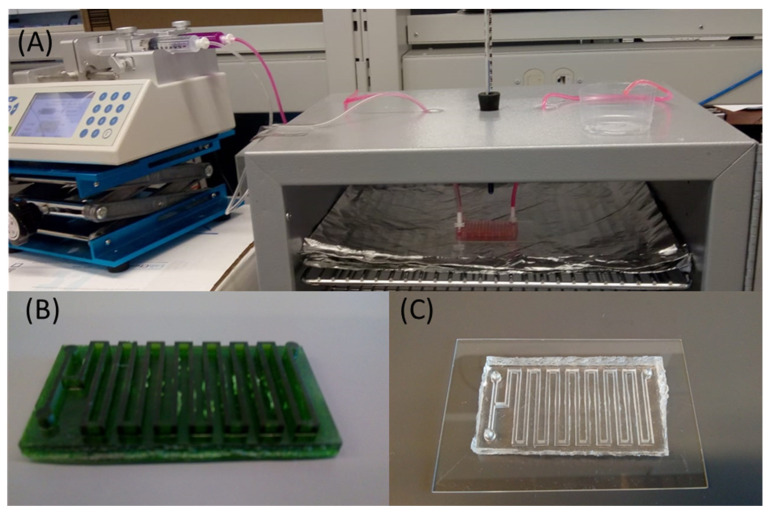
Operational set-up for microfluidic staining. (**A**) Microfluidic device placed inside oven with inlet and outlet tubings; (**B**) Photo of the mold used for PDMS casting; (**C**) Photo of the final bonded device.

As aforementioned, concentration and temperature are important parameters, thus their optimized values were adopted for the microfluidic device. When it comes to the flowing conditions, residency time becomes another important parameter that is subject to the external devices (i.e., syringe pump). In this paper, the total microchannel length was 400 mm, and its cross-sectional area was 2 × 2 mm. Using this design, we could achieve 5, 6, 7, 8, 9, 10, 11, and 12 min of residency time by applying corresponding flow rates of 7.82, 6.52, 5.58, 4.89, 4.34, 3.91, 3.55, and 3.26 µL/min, respectively. Note that the microfluidic device was placed inside the oven while the syringe pump was kept outside. The input and output hoses were long enough to enable sample collection and syringe manipulation outside the oven. Figure 5 shows the set-up arrangement.

**Figure 6:**
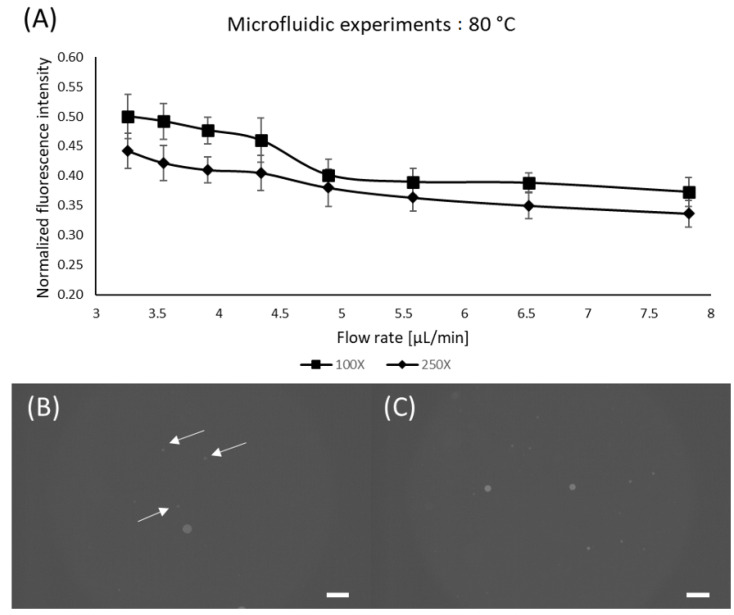
Microfluidic staining. (**A**) Effect of flow rate for fixed temperature; (**B**) PE microspheres stained using 100X Nile Red at 5.58 µL/min; (**C**) PE microspheres stained using 100X Nile Red at 3.26 µL/min; Scale bars are 100 µm. Arrows were added to indicate the particles of interest.

From the information acquired during the static experiments, we have performed the microfluidic experiments with the most promising configurations with respect to concentration and temperature (i.e., 100X and 250X; at 80 °C). Different flow rates were tested to compare the performance of static and microfluidic staining regarding the residency time. As expected, lower flow rates provided better results, which is in accordance with the static experiments [46,49]. Nonetheless, it is possible to observe that for the lowest flow rate (and highest residency time) the static staining had superior fluorescence levels (~37% higher). This behavior could be attributed to the lower mixing quality governed by diffusion inside the device since the static samples were actively shaken prior to oven insertion [50,51,52]. Even though the microfluidic results exhibited lower fluorescence levels compared to the static experiments, it provides passive mixing and staining without tedious and time-consuming manual sample preparation. Nonetheless, it is worth mentioning that for higher flow rates, identification becomes difficult due to low fluorescence levels arising from short residency time. Figure 6 shows the results for microfluidic staining of the PE microspheres.

**Figure 7:**
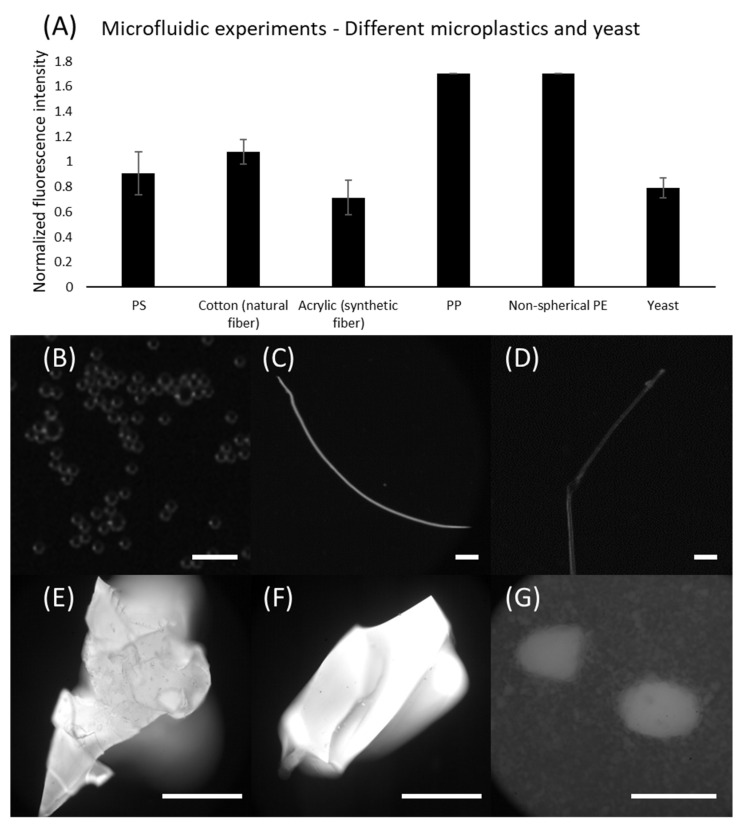
Microfluidic staining for different plastics and yeast. (**A**) Fluorescence levels for different microplastics and yeast; (**B**) PS microspheres; Scale bar is 50 µm; (**C**) Cotton (natural fiber); (**D**) Acrylic (synthetic fiber); Scale bars are 1 mm; (**E**) PP from storage container; Scale bars are 50 µm. (**F**) PE from storage container; Scale bars are 50 µm. (**G**) Yeast; Scale bars are 50 µm.

Besides PE microspheres, we further demonstrated the capabilities of our device for identifying other types of plastic. In this regard, multiple types of microplastics were applied, including microspheres (PS), fibers (cotton and acrylic), plastic parts scratched from storage containers (PP and PE). Moreover, yeast was adopted as a model of potential organic particles in seawater. Figure 7 shows the results of microfluidic staining for these samples. Note that PS microspheres showed better results when compared to the PE microspheres stained by the microfluidic device. Amongst the fibers, cotton indicated stronger fluorescence levels compared to acrylic, yet both were identifiable. Surprisingly, we found that all results obtained using PP and PE samples indicated the highest pixel intensity (i.e., 255, though larger than the threshold, it is indeed a strong indicator). However, as a recognized downside of staining identification, our method still suffers from the incapability of distinguishing microplastics from other natural particles, which can be seen from the results obtained using yeasts. It showed comparable fluorescence levels with respect to the plastics, highlighting the necessity for eliminating organic matter prior to sample analysis. Nevertheless, our results have demonstrated that continuous staining is achievable in microfluidic devices.

## 4. Discussion

In this paper, we have presented a novel microfluidic identification method for the continuous recognition of microplastics in water. Our method combines the Nile Red staining protocols with the high-throughput advantages imposed by microfluidics [45,47,51]. We acknowledge that the flow rates used must be small in order to achieve reasonable residency time, which has a negative effect on the throughput; however, the use of multiple (parallel) devices is feasible (especially due to its miniaturized size) which can enhance the throughput significantly [53,54]. In addition, the devices could be further improved and integrated into water monitoring stations in the future for continuous sampling and identification. According to the results obtained, the best staining quality is at the lowest flow rate (3.26 µL/min), which was expected since the static experiments showed that the lowest residency time performed the best.

In addition, though microfluidic results are still not as good as the static ones, future improvements can be carried out by adopting a better mixing strategy for Nile Red and samples [50,51,52]. Currently, a myriad of mixing methods has been developed for microfluidic devices, including both passive and active mixing. For example, better mixing performance could be addressed by adding pillars inside the channels [55,56]. Active mixers such as acoustofluidic mixers are alternatives and often provide more rapid mixing due to their superior particle control abilities [57].

The device can be further improved by coupling an on-chip heater, eliminating the need for an oven [58], thus reducing costs and enhancing its integrability. Once fully miniaturized, the device could be used for in situ analysis of water samples [47,48]. In situ analysis could also benefit from the use of smartphones, possibly for both identification and for device operation (pump and active mixers control) [59,60,61].

Note that the concentration of microplastics in seawater samples varies widely, being less concentrated off-shore (down to 8 particles/m³) [62]. In addition, global plastic distribution also changes significantly from one place to another, thereby a rapid and continuous identification prior to in-depth analysis would be beneficial. Though the staining method is not capable of distinguishing different types of microplastics, including other particles such as marine organisms, it indeed provides a simple, low-cost and effective method to confirm the presence of microplastics prior to more in-depth analysis including type differentiation [63]. Moreover, compared to regular visual inspection that bypasses the fluorescence staining, this proposed method turns microplastics into more prominent particles for better identification [64].

## 5. Conclusions

Overall, we have suggested the adoption of a microfluidic device for the continuous analysis and further detection of microplastics. Nile Red has proven to be effective for the identification of microplastics. Static experiments were performed to systematically assess the influence of staining agent concentration, temperature, and residency time. Based on the results, the microfluidic configuration for continuous staining was optimized, leading to the best fluorescence results among the tested configurations. Our method demonstrated to be feasible for the identification of different types of microplastics with the advantage of continuous staining and with the possibility of future integration for in situ identification along with higher throughputs. This platform demonstrated to successfully identify microplastics in a continuous manner, representing a valuable option for environmental management.

## Data Availability

Data are available from the corresponding author upon reasonable request.
